# Integrated transcriptomic and metabolomic analysis unveils biosynthetic mechanisms of key bioactive isoflavonoid accumulation in *Astragalus mongholicus* under different light treatments

**DOI:** 10.1093/hr/uhag192

**Published:** 2026-05-20

**Authors:** Zhen Wang, Panpan Wang, Xinxin Wang, Weili Liu, Jiao Xu, Weichao Ren, Xiubo Liu, Jian Wei, Wei Ma

**Affiliations:** Key Laboratory of Xinjiang Phytomedicine Resource and Utilization, Ministry of Education, College of Pharmacy, Institute for Safflower Industry Research, Shihezi University, Shihezi 832003, China; Pharmacy of College, Heilongjiang University of Chinese Medicine, Harbin 150040, China; Pharmacy of College, Heilongjiang University of Chinese Medicine, Harbin 150040, China; Pharmacy of College, Heilongjiang University of Chinese Medicine, Harbin 150040, China; Pharmacy of College, Heilongjiang University of Chinese Medicine, Harbin 150040, China; Pharmacy of College, Heilongjiang University of Chinese Medicine, Harbin 150040, China; Pharmacy of College, Heilongjiang University of Chinese Medicine, Harbin 150040, China; Pharmacy of College, Heilongjiang University of Chinese Medicine, Harbin 150040, China; College of Jiamusi, Heilongjiang University of Chinese Medicine, Jiamusi, China; Key Laboratory of Xinjiang Phytomedicine Resource and Utilization, Ministry of Education, College of Pharmacy, Institute for Safflower Industry Research, Shihezi University, Shihezi 832003, China; Pharmacy of College, Heilongjiang University of Chinese Medicine, Harbin 150040, China

## Abstract

Astragali Radix, the dried root of *Astragalus mongholicus*, is a prominent traditional Chinese medicinal material, with isoflavonoids serving as its primary bioactive components. Light serves as a pivotal environmental cue modulating isoflavonoid biosynthesis, its regulatory influence on *A. mongholicus* remains poorly understood, and the underlying biosynthetic pathways have yet to be fully elucidated. This study investigated the impacts of different light on isoflavonoid accumulation through integrated transcriptome and metabolome analyses. Compared to white light, red and blue light treatments significantly altered the expression of 93 genes within the isoflavonoid biosynthetic pathway, 52 of which were up-regulated. Metabolomic analysis revealed 35 differential isoflavonoid metabolites, with 23 compounds, including the primary medicinal components calycosin (CA) and formononetin (FO), showing increased accumulation. Notably, blue light exhibited a significantly stronger promoting effect on CA and FO accumulation than red light. Through integrated multi-omics analysis, we revealed the molecular network underlying this light quality–specific regulation. Correlation analysis revealed that multiple structural genes were significantly positively correlated with these key active components, among which *AmCHR*, *AmCHS*, *AmCHI*, and *AmIFS* were identified as core regulatory genes. The biochemical roles of candidate genes *AmCHR*, *AmCHS*, *AmCHI*, and *AmIFS* were validated via *in vitro* enzymatic assays. Furthermore, utilizing a newly established nonsterile hairy root transformation system, functional validation through overexpression and RNA interference experiments demonstrated that these candidate genes positively regulate the accumulation of key isoflavonoids, including CA, calycosin-7-O-β-D-glucoside, FO, and ononin. These findings were further corroborated by transient gene silencing using antisense oligodeoxynucleotides. This study elucidates the regulatory network of light-induced isoflavonoid biosynthesis in *A. mongholicus* and provides essential genetic resources for the metabolic engineering and sustainable production of these medicinal compounds.

## Introduction

*Astragalus mongholicus*, a perennial herb of the genus *Astragalus* (Fabaceae), possesses extremely high medicinal value [1]. Its dried roots serve as the traditional Chinese medicinal material Astragali Radix (Huangqi), which is widely applied in anti-inflammation, antibiosis, antitumor, cardioprotection, diabetes treatment, and immune enhancement [2]. The chemical constituents of *A. mongholicus* mainly include flavonoids, terpenoids, and polysaccharides [3]. Among them, isoflavonoids such as calycosin (CA), calycosin-7-O-β-D-glucoside (CAG), formononetin (FO), and ononin (ON) are its major bioactive components and constitute the primary indices for the phytochemical evaluation and grading of *A. mongholicus* [4]. Studies have shown that Astragali Radix potential anticoronavirus activity as a pharmaceutical agent. As a key constituent of Huashibaidu (HSBD) formula, it can be used in the treatment of COVID-19 [5]. Furthermore, Astragali Radix possesses the dual property of being both a medicinal herb and a foodstuff. In multiple countries and regions across America, Europe, and Asia, it is commonly used as an ingredient in daily beverages or soups and porridges to achieve health-promoting effects such as strengthening the body, lowering blood sugar and blood pressure, preventing influenza, and delaying aging [6]. As an important medicinal plant, *A. mongholicus* has extensive market demand. Consequently, enhancing both the biomass productivity and isoflavonoid accumulation in *A. mongholicus* is of paramount importance for its sustainable production and pharmaceutical efficacy.

As an essential environmental signal, light regulates the complete life cycle of plants [7]. It serves not only as the main energy supplier for photosynthesis but also as a key signal influencing photomorphogenesis and various physiological activities [8]. Solar radiation reaching the Earth’s surface covers a wavelength range from ~250 to 2500 nm, while the wavelengths that can affect plant photosynthesis and morphogenesis range roughly from ultraviolet (UV, 280 nm) to far-infrared (FIR, 700–750 nm) [9]. Within this spectrum, red and blue light are the primary drivers of photosynthesis and developmental transitions [10]. Specifically, red light irradiation is known to boost the storage of carbohydrates and hasten the progression of reproductive phases in plants [11]. Blue light can promote the development of plant root systems, enhance the thickness of main stems, and facilitate the synthesis of proteins and amino acids in plants [12]. The application of a red and blue light combination to tomato plants has been demonstrated to markedly elevate chlorophyll content and biomass yield, resulting in a substantial improvement in photosynthetic light-use efficiency [13].

Beyond primary growth, light serves as a fundamental regulator of plant secondary metabolism. A substantial body of research indicates that red and blue light wavelengths operate as critical signaling factors, which fine-tune the transcriptional activity of pathway-specific genes to coordinately regulate the production of specialized metabolites [14]. For example, exposure to red light has been shown to markedly increase the concentrations of total phenolic compounds, flavonoids, and the bioactive complex silymarin in callus cultures of *Silybum marianum* [15]. Optimal ratios of red to blue light, such as 9:1 or 7:3, have been reported to substantially enhance both root biomass production and flavonoid accumulation in certain plant systems [16]. It is important to note, however, that the effects of red and blue light spectra on secondary metabolic pathways often exhibit considerable species-specific variation. Specifically, blue light potently stimulates the accumulation of chicoric acid in *Taraxacum mongolicum*, while red light preferentially enhances the synthesis of other phenolic compounds like caffeine and chlorogenic acid [17]. In *Hypericum perforatum*, red light treatment significantly elevates the contents of hypericin and flavonoids, whereas blue light treatment exerts no significant effect [18]. These findings indicate that the regulatory effects of light quality are species-specific. Therefore, investigating the differential effects of blue and red light on medicinal isoflavonoid accumulation in *A. mongholicus* is of great significance for elucidating its specific regulatory mechanisms. Currently, enhancing the accumulation of bioactive components by modifying light conditions has become an effective approach to cultivating high-quality Chinese medicinal materials [19]. Therefore, elucidating the molecular mechanisms by which distinct light regimes drive the accumulation of key bioactive components, and optimizing light wavelengths for cultivation, holds substantial practical significance for industrial production.

Although the publication of the *A. mongholicus* genome has facilitated extensive research into the biosynthesis and regulatory mechanisms of its isoflavonoids [20], the specific impacts of light quality on its secondary metabolism remain largely unexplored. In addition, the genes encoding core enzymes in the isoflavonoid biosynthetic pathway have not been thoroughly characterized. Perhaps most limiting, the lack of an efficient genetic transformation platform in *A. mongholicus* has significantly hindered the functional verification of putative regulatory or biosynthetic genes. In this work, we examined the influence of red and blue light spectra on isoflavonoid production in *A. mongholicus* seedlings. By combining transcriptomic profiling with targeted metabolite analysis, we sought to unravel the molecular mechanisms underpinning the light-mediated accumulation of these compounds. This multi-omics integration enabled the identification of pivotal enzyme-encoding genes within the isoflavonoid biosynthetic network. *In vitro* experiments conclusively demonstrated the catalytic functions of the candidate enzyme genes *AmCHR*, *AmCHS*, *AmCHI*, and *AmIFS*, along with their specific roles in the isoflavonoid biosynthetic pathway of *A. mongholicus*. Furthermore, we unequivocally validated the *in vivo* biological function of these genes in promoting isoflavonoid synthesis using a newly established nonsterile hairy root genetic transformation system and antisense oligodeoxynucleotide (AsODN) technology.

## Results

### Red and blue light promote the biosynthesis of isoflavonoids in *A. mongholicus*

**Figure 1:**
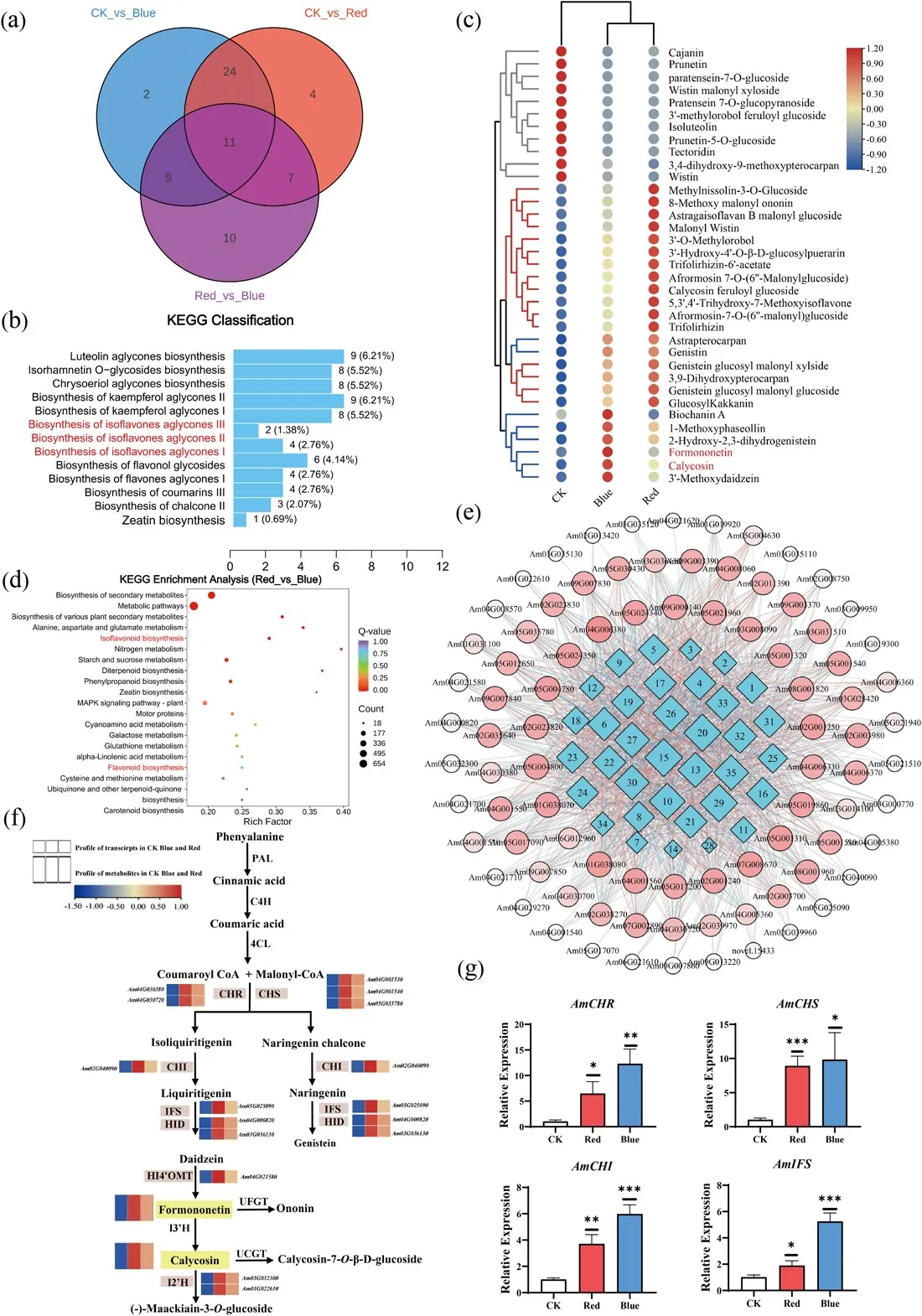
Multiomics analysis of red and blue light–regulated isoflavonoid biosynthesis in *A. mongholicus.* (a) Venn diagram illustrating the overlaps of DAMs among the different comparison groups. (b) KEGG pathway classification analysis comparing Red_ vs_ Blue. (c) Heatmap showing the accumulation patterns of 35 isoflavonoid DAMs in *A. mongholicus* across different light treatments. Dendrogram branches are color-coded: gray, red, and blue indicate the highest accumulation levels in the CK, red light, and blue light groups, respectively. (d) KEGG enrichment analysis of DEGs between Red and Blue groups. (e) Co-expression network of the 35 isoflavonoids and the genes involved in flavonoid and isoflavonoid biosynthesis. Red circular nodes represent DEGs, and blue diamond-shaped nodes represent isoflavonoid DAMs. Node size represents connectivity, with larger nodes indicating higher connectivity. Line thickness represents the absolute value of the correlation coefficient, with thicker lines indicating stronger correlations. Red lines indicate positive correlations, and blue lines indicate negative correlations. (f) Relative expression patterns of the annotated biosynthetic genes within the isoflavonoid pathway that exhibit positive correlations with metabolites. (g) qRT-PCR analysis of the candidate genes. Error bars represent the mean ± standard deviation (SD) of three technical replicates. ^*^P < 0.05, ^**^  *P* < 0.01, and ^***^  *P* < 0.001.

To investigate the impact of light quality on isoflavonoid accumulation in *A. mongholicus*, hydroponically grown seedlings were exposed to white light (CK), red light, or blue light for 30 days, followed by comprehensive metabolomic profiling. A total of 2153 metabolites were identified, among which flavonoids were the most abundant category (473 compounds), including 92 isoflavonoids. Venn diagram analysis of differentially accumulated isoflavonoids identified 67 differential metabolites: 46 in Red _ vs_ CK, 37 in Red _ vs_ Blue, and 46 in Blue_ vs_ CK, with 11 common differential metabolites shared across all comparison groups. Between the CK_ vs_ Red and CK_ vs_ Blue groups, 57 differential isoflavonoid metabolites were detected, among which 35 were common differential metabolites (Fig. 1a). KEGG classification demonstrated that the differentially accumulated metabolites (DAMs) were predominantly enriched in the ‘biosynthesis of isoflavones aglycones I’, ‘biosynthesis of isoflavones aglycones II’, and ‘biosynthesis of isoflavones aglycones III’ pathways (Fig. 1b). Further analysis revealed that both blue and red light significantly enhanced the accumulation of 23 isoflavonoids, including the key bioactive constituents CA and FO. Notably, the levels of CA and FO under blue light were significantly higher than those observed under red light treatment (Fig. 1c). These results indicate that both blue and red light can enhance the biosynthesis of isoflavonoids in *A. mongholicus*, with blue light exhibiting a stronger promoting effect.

Transcriptome data from the identical samples were also subjected to systematic analysis. A total of 42 013 transcripts were annotated, and the mapping rates for total reads mapped and uniquely mapped reads to the reference genome were all above 80% across all experimental groups. Volcano plot analysis identified 12 493 differentially expressed genes (DEGs) in the CK_ vs_Blue comparison, consisting of 5622 up-regulated and 6871 down-regulated genes. The CK_ vs_ Red light comparison yielded 12 850 DEGs, including 5960 up-regulated and 6890 down-regulated genes. In the Red_ vs_ Blue comparison, 7353 DEGs were identified, with 3792 up-regulated and 3561 down-regulated genes (Fig. S1A). Principal component analysis (PCA) was performed based on FPKM (Fragments Per Kilobase of transcript per Million mapped reads). Principal components PC1, PC2, and PC3 accounted for 48.77%, 27.11%, and 4.83% of the total variance, respectively (Fig. S1B). Notably, red light samples formed distinct clusters separate from those of blue and white light. Among the up-regulated DEGs under blue and red light, the most significantly enriched KEGG pathways included ‘phenylpropanoid biosynthesis’ (ko00940), ‘isoflavonoid biosynthesis’ (ko00943), and ‘flavonoid biosynthesis’ (ko00941) (Fig. 1d).

To investigate the reprogramming regulatory mechanisms underlying isoflavonoid secondary metabolism in *A. mongholicus*, this study first analyzed the enriched pathways of DEGs and screened a candidate gene set involved in metabolite biosynthesis. Nevertheless, the functional interrelationships between these metabolism-related genes and their potential regulators require further clarification. To further dissect the regulatory influence of red and blue light on the isoflavonoid pathway, a gene–metabolite co-expression network was constructed based on Pearson correlation coefficients between the DEGs and DAMs (Fig. 1e). In this network, transcripts and metabolites serve as nodes, and their correlations as edges, revealing their synergistic relationships *in vivo* through the correlation of expression abundance between nodes. The dataset used in this study was obtained from the CK_vs_Red and CK_vs_Blue comparisons, which together identified 35 isoflavonoid DAMs and 93 DEGs (Supplementary Table S1) enriched in the flavonoid and isoflavonoid biosynthetic pathways. Numbers 1–35 within nodes represent the 35 differentially accumulated isoflavonoid metabolites, with corresponding compound IDs and detailed information provided in Supplementary Table S2. Analysis revealed that these DEGs and DAMs exhibited robust positive or negative correlations, with absolute correlation coefficients |r| > 0.9 in all cases. Among them, 17 DEGs (*Am01G022610*, *Am01G031100*, *Am02G040090*, *Am03G000770*, *Am03G036130*, *Am04G000820*, *Am04G001530*, *Am04G001540*, *Am04G008570*, *Am04G021580*, *Am04G030380*, *Am04G030700*, *Am04G030720*, *Am05G032300*, *Am05G035780*, *Am09G013220*, and *Am05G025090*) showed significant positive correlations with the key medicinal constituents of *A. mongholicus*, CA and FO, highlighting their potential roles in light-mediated isoflavonoid biosynthesis.

To gain deeper insights into the potential biological functions of these genes associated with isoflavonoid biosynthesis, the 17 genes that showed positive correlations with CA and FO in the co-expression network analysis were annotated using the Pfam and NCBI databases. Functional annotation revealed that 12 of these DEGs encoded enzymes involved in the isoflavonoid biosynthetic pathway. These included 2 *CHR* (chalcone reductase), 3 *CHS* (chalcone synthase), 1 *CHI* (chalcone isomerase), 1 *IFS* (isoflavone synthase), 2 *HID* (2-hydroxyisoflavanone dehydratase), 2 *I2′H* (isoflavone 2′-hydroxylase), and 1 *HI4′OMT* (isoflavone 4′-O-methyltransferase) genes. The expression patterns of these candidate genes and the changes in the content of key medicinal constituents were analyzed. A biosynthetic pathway map of isoflavonoids in *A. mongholicus* was constructed, which preliminarily elucidated the potential functions of these genes (Fig. 1f). Notably, the expression patterns of *AmCHR* (Am04G030380), *AmCHS* (Am04G001530), *AmCHI* (Am02G040090), and *AmIFS* (Am05G025090) were highly consistent with the accumulation trends of CA and FO, showing significant positive correlations, indicating that these genes may function as core rate-limiting enzymes in light-regulated isoflavonoid biosynthesis. Therefore, we selected these four genes as candidates for further functional validation through *in vitro* and *in vivo* experiments. Furthermore, quantitative reverse-transcription PCR (qRT-PCR) analysis corroborated that the expression trends of these candidate genes were highly consistent with the transcriptomic data, thereby validating the reliability of the RNA-seq results (Fig. 1g).

### Gene cloning and subcellular localization analysis

**Figure 2:**
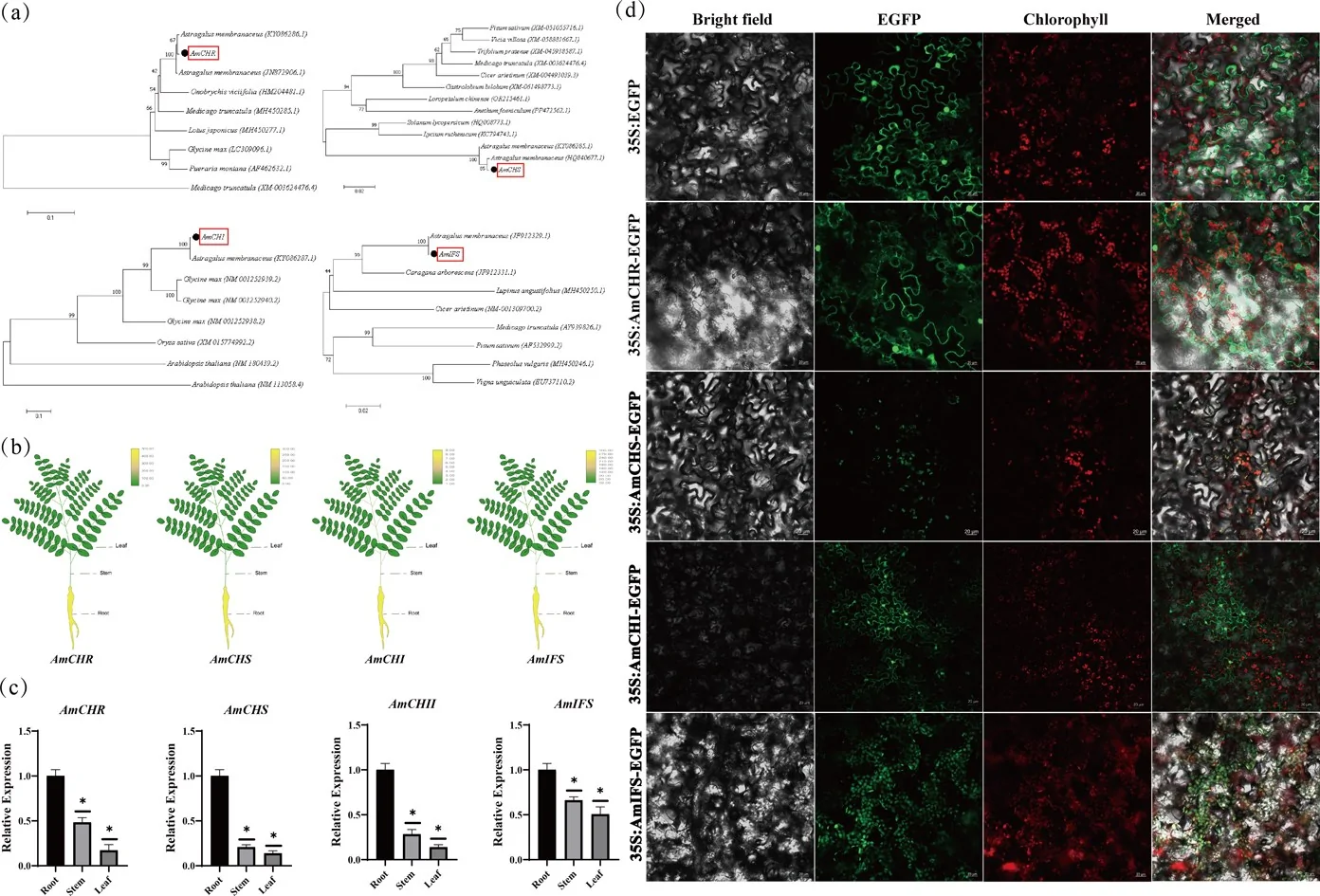
Classification and expression pattern analysis of candidate genes. (a) Phylogenetic tree of the candidate genes and their homologous genes from other species. (b) Subcellular localization of the candidate genes in *Nicotiana benthamiana* leaves. Scale bar = 20 μm. Excitation wavelengths were 488 nm for EGFP and 680 nm for chlorophyll autofluorescence. (c) Heat map of candidate gene expression levels in different tissues. (d) qRT-PCR analysis of candidate gene expression levels in different tissues. Error bars represent the mean SD of three technical replicates. ^*^P < 0.05, ^**^P < 0.01 and ^***^P < 0.001.

Based on transcriptomic data, we cloned and sequenced the full-length open reading frames (ORFs) of the candidate genes *AmCHR*, *AmCHS*, *AmCHI*, and *AmIFS* (Supplementary Table S3). Sequence analysis revealed that the deduced amino acids of these genes exhibited high homology with their counterparts in *A. membranaceus*, consistent with the taxonomic classification of *A. mongholicus* as a variety of *A. membranaceus* (Fig. 2a). Subcellular localization assays revealed that the enhanced green fluorescent protein (EGFP) control was detected in both the cytoplasm and nucleus, whereas the AmCHR-EGFP and AmCHI-EGFP fusion proteins were localized exclusively to the nucleus and cytoplasm, and the AmIFS-EGFP and AmCHS-EGFP fusion proteins were specifically targeted to chloroplasts (Fig. 2b), consistent with their predicted localizations. The expression patterns of genes across different tissues can provide important clues to their functions. Integrated results from transcriptomic data (Supplementary Table S4) and qRT-PCR analysis revealed that *AmCHR*, *AmCHS*, *AmCHI*, and *AmIFS* were constitutively expressed in the roots, stems, and leaves, with their highest abundance observed in the roots (Fig. 2c and d).

### Protein characterization and identification of catalytic functions

**Figure 3:**
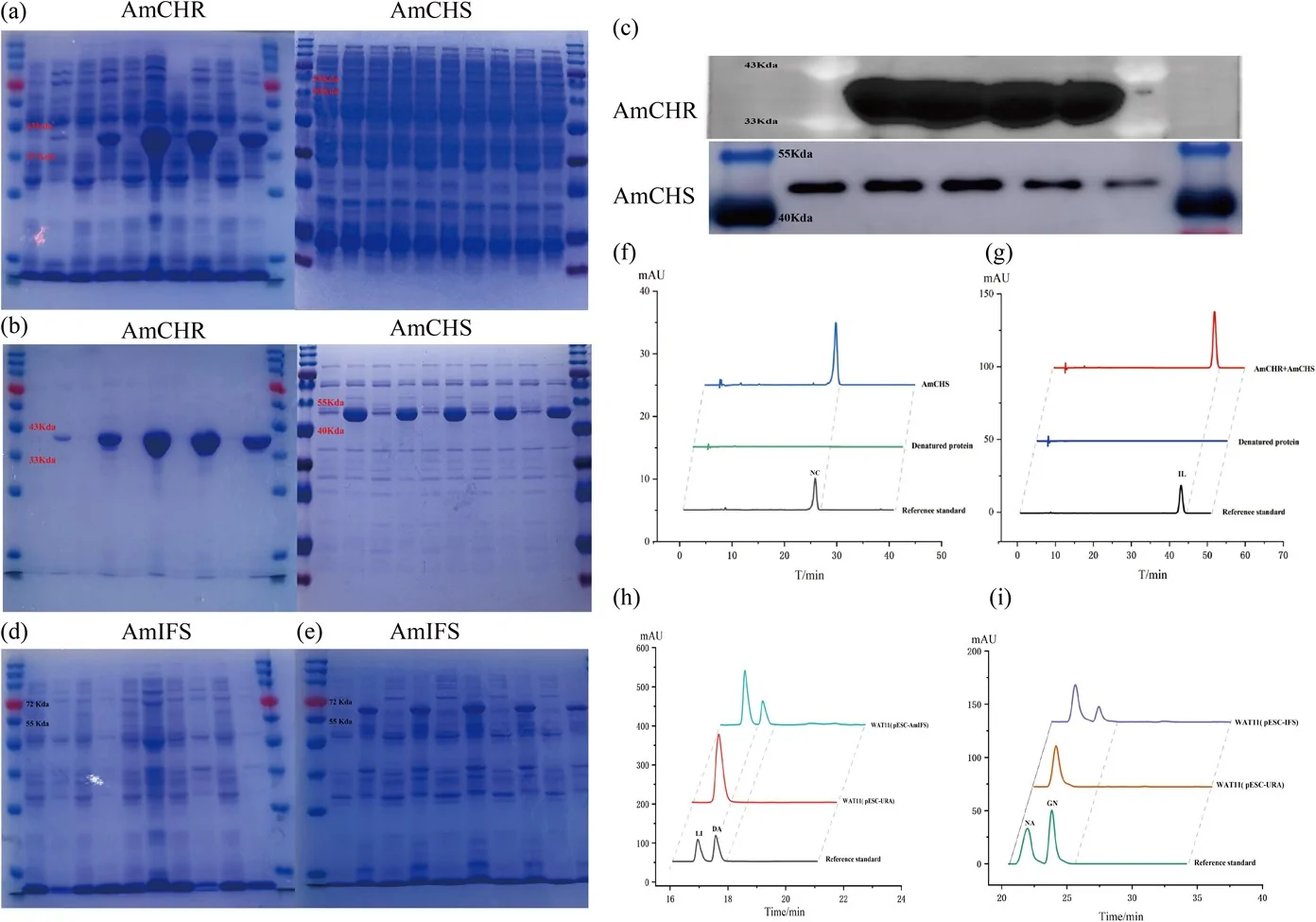
Characterization and catalytic function identification of proteins encoded by candidate genes. (a) SDS-PAGE analysis of crude protein extracts of AmCHR and AmCHS under different temperature induction conditions. (b) SDS-PAGE analysis of purified AmCHR and AmCHS proteins induced at different temperatures. (c) Detection of AmCHR and AmCHS proteins by WB. (d) SDS-PAGE analysis of crude protein expressing *AmIFS* gene. (e) SDS-PAGE analysis of cell lysates expressing *AmIFS*. (f) HPLC analysis of the catalytic function of the AmCHS protein. NC, naringenin chalcone. (g) HPLC analysis of the catalytic function of the AmCHS and AmCHR protein. IL, isoliquiritigenin. (h) HPLC chromatograms of the products derived from liquiritigenin catalyzed by recombinant AmIFS. LI, liquiritigenin; DA, daidzein. (i) HPLC chromatograms of the products derived from naringenin catalyzed by recombinant AmIFS. NA, naringenin; GN, genistein.

To assess the catalytic functions of the candidate proteins, the recombinant constructs pET28a-AmCHR, pET28a-AmCHS, and pET28a-AmCHI were individually transformed into *Escherichia coli* BL21(DE3) cells for heterologous expression. Recombinant protein expression was induced by adding isopropyl β-D-1-thiogalactopyranoside (IPTG) to a final concentration of 0.5 mM. A temperature gradient was tested to identify the optimal condition for soluble protein production. Following protein extraction and purification, sodium dodecyl sulfate–polyacrylamide gel electrophoresis (SDS-PAGE) analysis was performed. The induction and catalytic functions of the AmCHI protein have been previously published by our research group [21]. The crude protein SDS-PAGE gel results showed that all samples contained numerous endogenous protein bands. A prominent band for AmCHR was observed between 33 and 43 kDa, and a prominent band for AmCHS was observed between 40 and 55 kDa (Fig. 3a). To further identify the expressed proteins, purification was performed using the His-tag, followed by loading and detection. Purified SDS-PAGE results displayed highly homogenous bands for AmCHR and AmCHS consistent with their theoretical molecular masses (Fig. 3b). All bands corresponded to the predicted sizes of the proteins encoded by the AmCHR and AmCHS genes. These findings indicate that AmCHR and AmCHS were successfully induced in *E. coli* BL21 and exhibited high solubility in the supernatant. To verify the identity of the recombinant proteins, western blot (WB) analysis was performed using an anti-His antibody, which further confirmed their successful expression (Fig. 3c). However, no protein band corresponding to AmIFS was detected within the 55–72 kDa range (Fig. 3d). Subsequently, SDS-PAGE analysis of the lysed bacterial cells revealed a target protein band within the 55–72 kDa range, indicating that the expressed AmIFS fusion protein was present in the form of inclusion bodies (Fig. 3e). Notably, when the induction temperature was set at 25°C, the expression levels of AmCHR, AmCHS, and AmIFS proteins peaked, being significantly higher than those under other temperature conditions.

To verify the catalytic activities of the candidate enzymes, *in vitro* enzymatic assays were conducted using purified recombinant AmCHS and AmCHR, with heat-denatured proteins serving as negative controls. The *in vitro* reaction system was established employing p-coumaroyl-CoA and malonyl-CoA as substrates. The results showed that when only AmCHS protein was added to the system, naringenin chalcone was produced (Fig. 3f). In contrast, the simultaneous addition of equimolar AmCHS and AmCHR, supplemented with 1 mM nicotinamide adenine dinucleotide phosphate (NADPH), resulted in the formation of isoliquiritigenin (Fig. 3g). As *AmIFS* belongs to the cytochrome P450 family, its expression in *E. coli* is inefficient and seldom yields soluble protein; therefore, the *Saccharomyces cerevisiae* expression system was selected in this study to characterize its catalytic function. The AmIFS gene was cloned into the binary plasmid pESC-URA and heterologously expressed in the yeast strain WAT11. After separately adding the substrates liquiritigenin and naringenin to the culture of the recombinant yeast (Fig. 3h), the products daidzein and genistein were detected in the culture extracts (Fig. 3i). LC-MS/MS analysis further confirmed the identity of these products. When liquiritigenin was supplied as the substrate, the reaction product showed retention time and MS/MS fragmentation pattern identical to those of the authentic daidzein standard. When naringenin was supplied as the substrate, the reaction product showed retention time and MS/MS fragmentation pattern identical to those of the authentic genistein standard (Fig. S3). It is well established that IFS catalyzes the conversion of flavanones to 2-hydroxyisoflavanones, which are unstable and undergo spontaneous dehydration to form the corresponding isoflavones. The intermediate 2-hydroxyisoflavanone was not detected in our assay, which is consistent with its rapid non-enzymatic dehydration under the experimental conditions. Therefore, the detection of daidzein and genistein as the final products confirms that AmIFS encodes a functional isoflavone synthase capable of converting both liquiritigenin and naringenin to their respective isoflavones. This substrate promiscuity highlights the potential significance of this enzyme in the isoflavonoid biosynthetic pathway.

### *Agrobacterium rhizogenes*–mediated genetic transformation of *A. mongholicus* under nonsterile conditions

**Figure 4:**
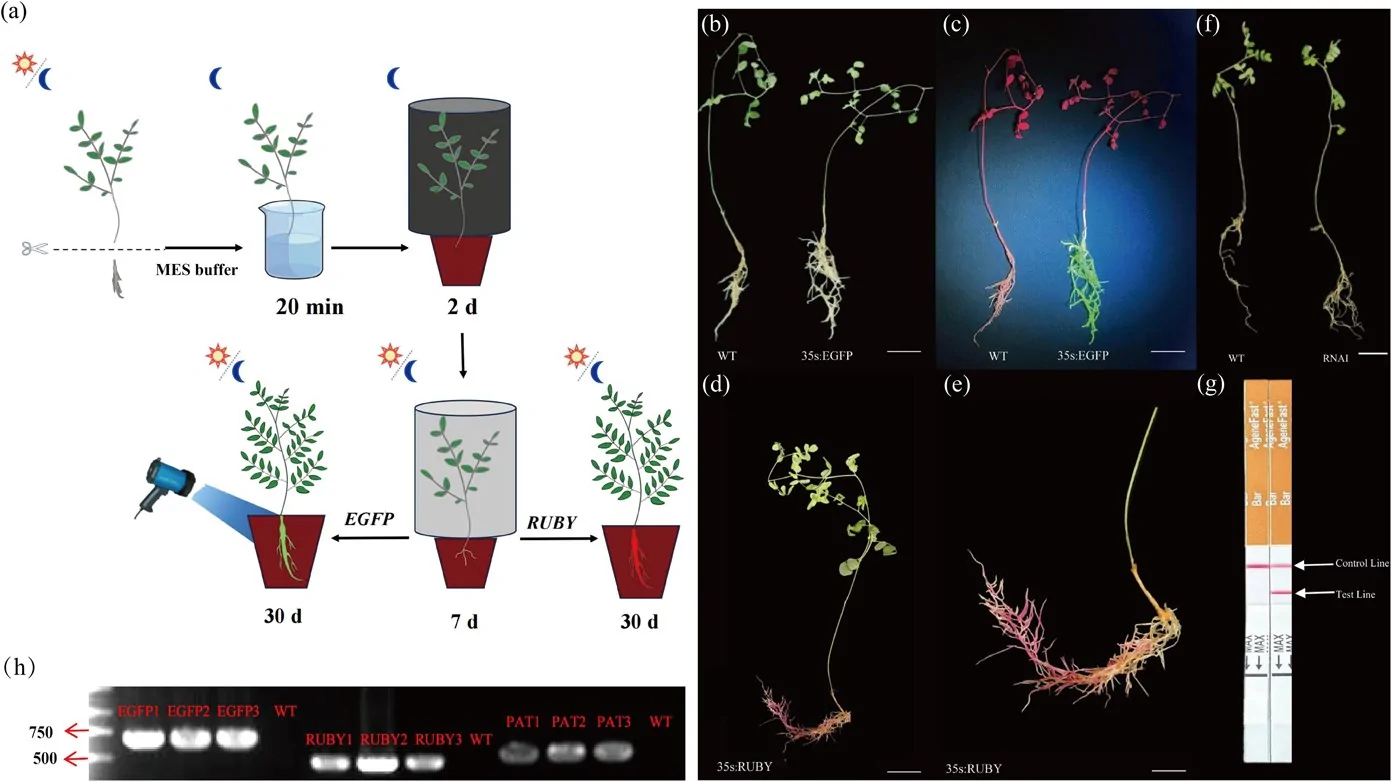
Construction of a genetic transformation system for *A. mongholicus* using *A. rhizogenes.* (a) Flowchart of the *A. rhizogenes*–mediated genetic transformation system for *A. mongholicus*. (b) WT and transgenic lines harboring the *EGFP* reporter gene under normal light conditions. (c) WT and transgenic lines harboring the *EGFP* reporter gene under blue excitation light. (d and e) Transgenic hairy roots harboring the *RUBY* reporter gene. (f) WT and transgenic lines harboring the *PAT* reporter gene under normal light conditions. (g) Bar strip assay for WT and transgenic hairy roots harboring the *PAT* reporter gene. (h) PCR detection confirmed that the hairy roots positive for *EGFP*, *RUBY*, and *PAT* were transgenic positive lines.

Genetic transformation of medicinal plants remains formidable and time-consuming; consequently, successful genetic engineering has been achieved in only a limited number of medicinal species to date. To overcome the recalcitrance of *A. mongholicus* to conventional transformation and evaluate the feasibility of *A. rhizogenes*–mediated delivery, seedlings were utilized as the primary explants in this study. This study established a novel method to generate transgenic hairy roots in *A. mongholicus* without requiring sterile conditions or complex tissue culture procedures (Fig. 4a). Twenty-one-day seedlings were cut at the stem–root junction under nonsterile conditions, with the hypocotyl portion retained (Fig. S2a). The wounded sites were subsequently inoculated with *A. rhizogenes* strain K599 harboring vectors containing *EGFP*, *RUBY*, or *PAT* reporter genes. The inoculated seedlings were planted in moist vermiculite, incubated in darkness for 2 days, and then transferred to standard photoperiod conditions for continued growth. Owing to the root inducing plasmid carried by *A. rhizogenes*, hairy roots began to form ~2 weeks later (Fig. S2b). After 30 days of culture, hairy roots were sampled and identified. Positive hairy roots carrying the *EGFP* reporter gene exhibited green fluorescence under blue excitation light, whereas wild-type (WT) hairy roots showed no fluorescence (Fig. 4b and c). Transgenic carrying the *RUBY* reporter gene produced a visually detectable red color (Fig. 4d and e). Transgenic expressing the *PAT* reporter gene caused the Bar test strip to display two purplish red bands in both the test and control zones, while WT hairy roots resulted in only one band in the control zone (Fig. 4f and g).

To molecularly verify the stable genomic integration of the transgenes, genomic DNA was isolated from putative transgenic hairy root lines. PCR amplification was then performed employing gene-specific primers designed for the *EGFP*, *RUBY*, and *PAT* reporter sequences. The results showed that bands corresponding to the respective reporter genes were successfully amplified from the positive hairy roots, and their sizes matched the expected target genes, while no such bands were amplified from the WT (Fig. 4h). These results collectively indicate that the reporter gene cassettes mediated by *A. rhizogenes* strain K599 were stably integrated into the genome of *A. mongholicus*, thereby effectively inducing the formation of transgenic hairy roots. This outcome substantiates the successful development of a robust and efficient genetic transformation protocol for *A. mongholicus* hairy roots under nonsterile conditions. Subsequent to transformation with the three distinct reporter constructs, both the survival rate of infected explants and the final transformation efficiency were quantified. Statistical analysis revealed that the survival rates across all treatment groups consistently exceeded 90%. The results showed that the plant survival rates all exceeded 90%. Specifically, the transformation rates were 75% for *EGFP* reporter gene, 26.7% for *RUBY* reporter gene, and 66.7% for *PAT* reporter gene (Supplementary Table S5). These data indicate that the transformation system achieved high plant survival and positive transformation rates. In conclusion, we have successfully implemented an efficient and reproducible non-sterile hairy root transformation platform for *A. mongholicus*. This system provides a robust and reliable *in vivo* platform for the functional characterization of candidate genes in this medicinally important species.

### Candidate gene expression significantly impacts isoflavonoid biosynthesis

**Figure 5:**
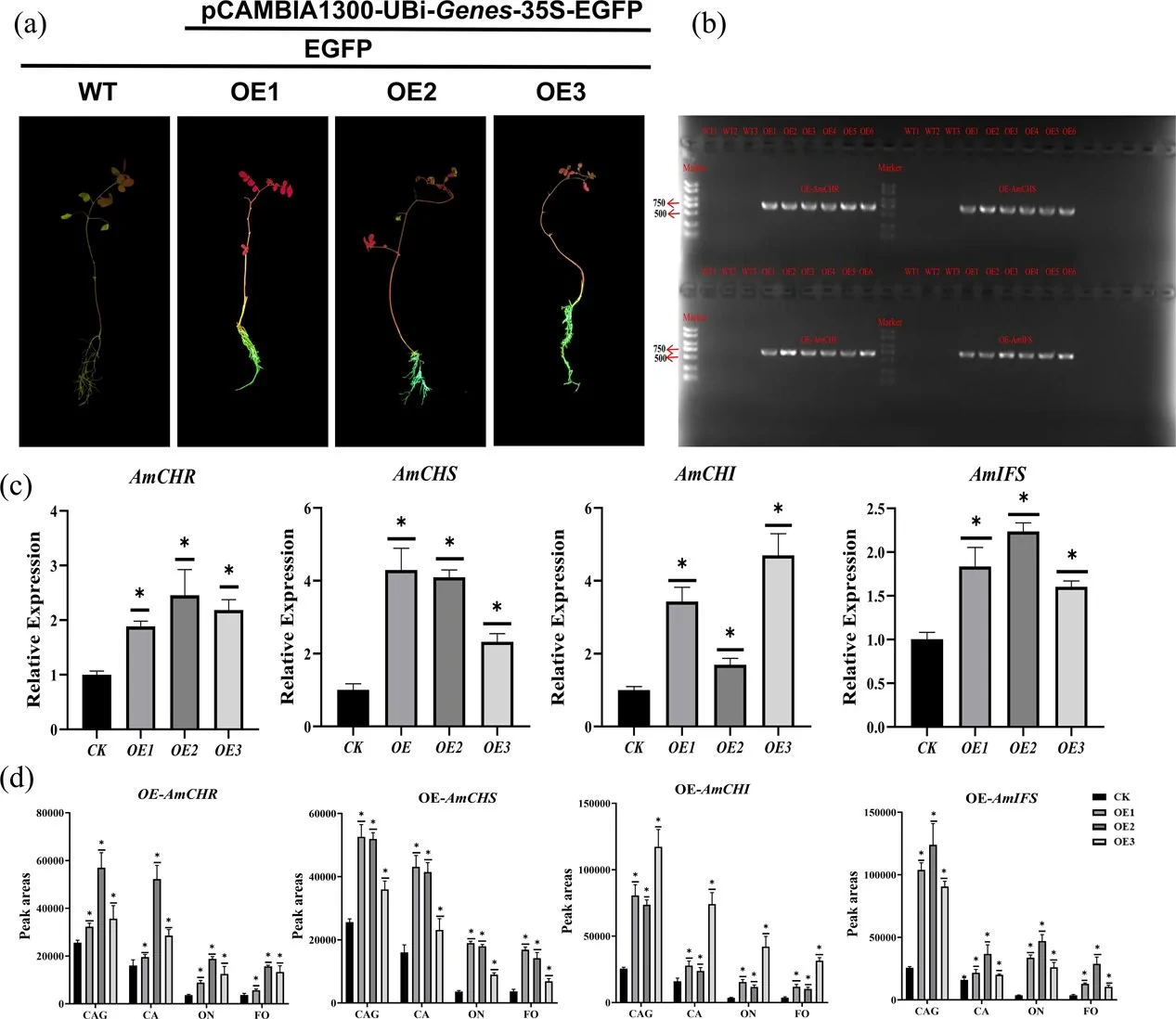
Overexpression of *AmCHR*, *AmCHS*, *AmCHI*, and *AmIFS* genes promoted the accumulation of isoflavonoids in *A. mongholicus*. (a) Green fluorescence signals of positive transgenic hairy roots under blue light excitation. (b) Molecular identification of transgenic hairy roots by PCR. (c) Relative transcript levels of the candidate genes in the OE lines determined by qRT-PCR. (d) Accumulation of major isoflavonoids in the OE lines. OE1, OE2, and OE3 represent three independent transgenic lines. Error bars represent mean ± SD of three technical replicates. ^*^P < 0.05.

**Figure 6:**
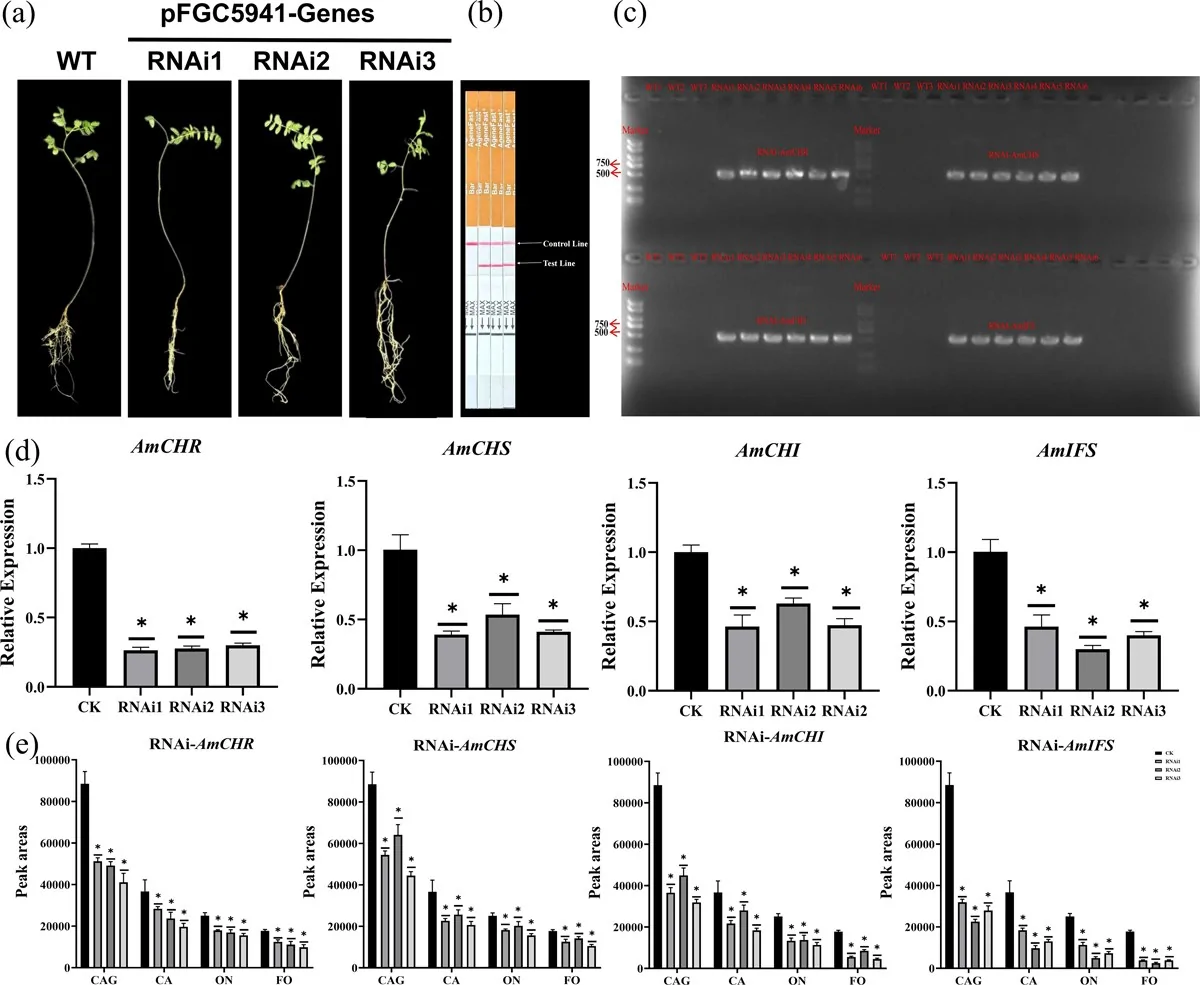
RNAi of *AmCHR*, *AmCHS*, *AmCHI*, and *AmIFS* genes inhibited the accumulation of isoflavonoids in *A. mongholicus*. (a) Phenotype of WT and RNAi hairy root lines. (b) Immunochromatographic Bar-strip assay for the rapid detection of transgenic hairy roots. (c) Molecular identification of transgenic lines by PCR. (d) Relative expression levels of candidate genes in RNAi lines. (e) Contents of isoflavonoids in RNAi lines. RNAi1, RNAi2, and RNAi3 denote three independent RNAi lines. Error bars represent mean ± SD of three technical replicates. ^*^P < 0.05.

Using the established nonsterile hairy root genetic transformation system for *A. mongholicus*, overexpression transgenic lines of the candidate genes were generated. Primary screening was performed by observing green fluorescence under blue light excitation (Fig. 5a), followed by PCR amplification targeting the *EGFP* reporter gene for confirmation (Fig. 5b). Ultimately, positive overexpression (OE) transgenic hairy roots of *AmCHR*, *AmCHI*, *AmCHS*, and *AmIFS* were successfully obtained. qRT-PCR analysis confirmed that the transcript levels of the target genes in all OE lines were significantly elevated relative to the CK (empty vector-transformed hairy roots) controls (Fig. 5c). Similarly, RNA interference (RNAi) transgenic lines of the candidate genes were generated using the same system. Primary screening was conducted using Bar test strips (Fig. 6a and b), with subsequent confirmation by PCR amplification targeting the *PAT* reporter gene (Fig. 6c). Consequently, stable RNA interference (RNAi) hairy root lines were successfully generated for each of the four target genes: *AmCHR*, *AmCHI*, *AmCHS*, and *AmIFS* (Fig. 6d).

The contents of CA, CAG, FO, and ON were further measured in the transgenic lines. Compared to the CK, overexpression of *AmCHR*, *AmCHI*, *AmCHS*, and *AmIFS* significantly enhanced isoflavonoid accumulation, with the AmCHI-OE and AmIFS-OE lines exhibiting the most prominent increases (Fig. 5d). Conversely, the isoflavonoid contents in the RNAi lines of each gene were significantly decreased, and again, the reductions in *AmCHI* and *AmIFS* lines were the most substantial (Fig. 6e). These results indicate that red and blue light signaling promotes isoflavonoid biosynthesis in *A. mongholicus* by upregulating the expression of *AmCHR*, *AmCHI*, *AmCHS*, and *AmIFS*. The more pronounced effects of *AmCHI* and *AmIFS* on isoflavonoid accumulation suggests that they function as pivotal rate-limiting enzymes in the isoflavonoid biosynthetic pathway.

### Experimental validation of candidate genes regulating isoflavonoid biosynthesis in *A. mongholicus* leaves via AsODN

**Figure 7:**
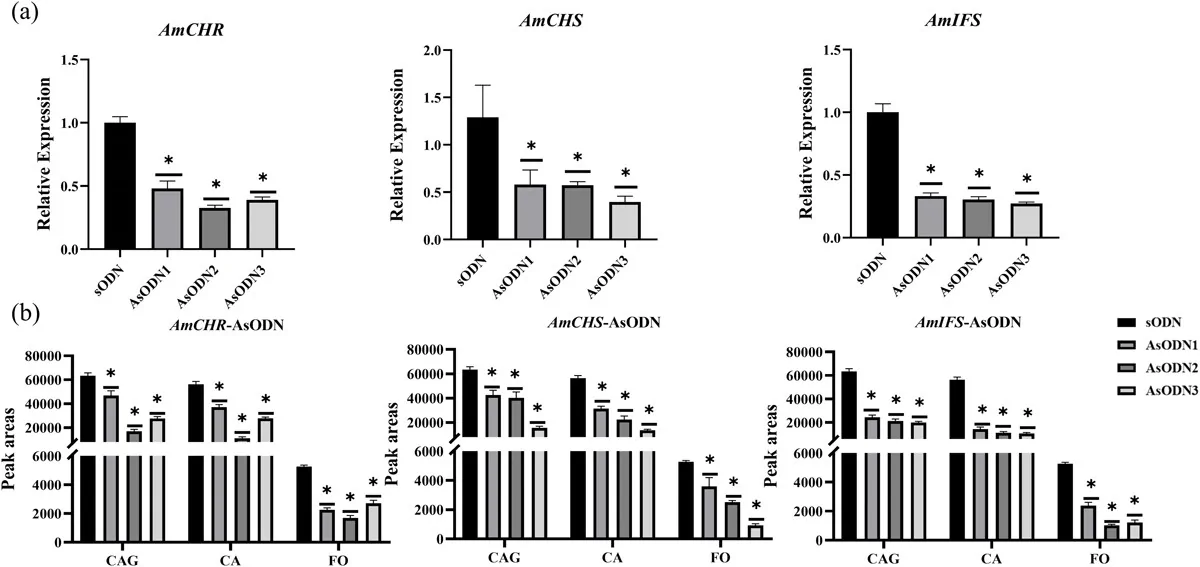
Functional validation via AsODNs reveals the role of candidate genes in isoflavonoid biosynthesis in *A. mongholicus* leaves. (a) Expression levels of candidate genes under AsODN treatment. (b) Changes in isoflavonoid contents in *A. mongholicus* leaves caused by transient inhibition of candidate genes. sODN treatment served as the control group, and AsODN1, AsODN2, and AsODN3 represented three independent leaf samples from individual plants. Error bars represent mean ± SD of three technical replicates. ^*^P < 0.05.

AsODN inhibit gene expression by specifically binding to target mRNA. In this study, AsODN technology was used to transiently silence the candidate genes *AmCHR*, *AmCHS*, and *AmIFS* in leaves of *A. mongholicus* and to analyze their effects on isoflavonoid biosynthesis (AsODN results for *AmCHI* have been published previously). Leaves were collected 48 h after treatment and analyzed by qRT-PCR and LC-MS. Compared with sense oligonucleotide (sODN) controls, AsODN treatment significantly reduced the expression of *AmCHR*, *AmCHS*, and *AmIFS* (Fig. 7a). Gene downregulation further led to significant changes in isoflavonoid accumulation in leaves, with marked decreases in the contents of CAG, CA, and FO (Fig. 7b). These *in vivo* findings align closely with the results of the metabolic analysis conducted in the RNAi hairy root system. Collectively, this evidence substantiates the conclusion that *AmCHR*, *AmCHS*, *AmCHI*, and *AmIFS* play pivotal roles in the isoflavonoid biosynthetic pathway of *A. mongholicus*.

## Discussion

Light is an indispensable environmental factor that profoundly influences plant life cycles, playing a dual role in modulating both morphogenesis and the biosynthesis of specialized metabolites [22]. Growing evidence suggests that blue and red light wavelengths can act as signaling elicitors, regulating the expression of pathway genes within plants and thereby modulating the synthesis of secondary metabolites [23]. For instance, blue and red light induce the expression of the transcription factor FtMYB116 in tartary buckwheat, which, in turn, directly activates *F3'H* expression, ultimately promoting flavonoid biosynthesis [24]. Similarly, blue light treatment upregulates key flavonoid biosynthetic genes, such as *Es4CL1*, *EsCHS2*, and *EsCHI1* in *Epimedium sagittatum*, leading to increased levels of epimedin C, icariin, and icariside II [25]. Supplementary lighting with a blue-to-red ratio of 1:3 significantly increased the content of indigo and indirubin, the major bioactive metabolites in *Isatis indigotica* [26]. However, the regulatory effects of light treatment on secondary metabolites vary among different plant species. In the present study, we evaluated the effects of white, red, and blue light on isoflavonoid accumulation in the roots of *A. mongholicus*. Our results demonstrated that both red and blue light stimulated the biosynthesis of 23 isoflavonoids, most notably CA and FO, with blue light exhibiting a more potent inductive effect than red light. This finding is consistent with the species-specific nature of light quality regulation, further confirming the species-dependent nature of light quality regulation. This differential response may be attributed to differences in the expression patterns of photoreceptors and their downstream signal transduction pathways among different species, with photoreceptors such as cryptochromes and phototropins potentially playing distinct regulatory roles in different species. These photoreceptors have been extensively characterized in model plants and shown to regulate secondary metabolite biosynthesis in response to blue light [27]. They likely activate downstream transcription factors, including HY5 and MYB family members, which are known regulators of flavonoid biosynthetic genes in various plant species. Consistent with this, promoter analysis of the four core genes revealed multiple light-responsive *cis*-elements and MYB binding sites in their upstream regions (Fig. S4, Supplementary Table S6), suggesting that they may be direct targets of light signaling and MYB transcription factors. The stronger promoting effect of blue light observed in this study suggests that blue light may serve as a common elicitor for secondary metabolism in medicinal plants, although the specific regulatory mechanisms may vary among species [28], highlighting the importance of species-specific optimization for light-based cultivation strategies.

In recent years, the rapid evolution of high-throughput sequencing and multi-omics integration has established large-scale data analysis as a cornerstone for identifying novel genes involved in specialized metabolite biosynthesis [29]. Preliminary integrated multi-omics analyses of the isoflavonoid biosynthetic pathway have been conducted in various leguminous medicinal plants, including *Iris domestica* [30], *Glycyrrhiza glabra* [31], and *Trifolium pratense* [32]*.* These studies have enriched the genomic resources for isoflavonoid biosynthesis, with genes such as *CHR*, *CHS*, and *IFS* being widely demonstrated as key enzyme genes in the pathway [33]. By integrating transcriptomic and targeted metabolomic datasets from *A. mongholicus* roots subjected to varying light qualities, we constructed a robust gene-metabolite co-expression network to model the specific isoflavonoid biosynthetic architecture. Among these, 17 DEGs involved in the isoflavonoid biosynthetic pathway showed significant positive correlations with the contents of CA and FO. Twelve of these DEGs encoded key enzyme genes in the pathway, specifically including two *CHR*, three *CHS*, one *CHI*, one *IFS*, two *HID*, two *I2'H*, and one *HI4'OMT*. While this network represents an initial step toward fully deciphering the light-mediated regulation of isoflavonoids, it provides essential predictive insights into the core enzymatic machinery. In this network, *AmCHR* (Am04G030380), *AmCHS* (Am04G001530), *AmCHI* (Am02G040090), and *AmIFS* (Am05G025090) were identified as core genes, suggesting their potential roles as rate-limiting steps in the pathway.

Chalcone reductase (CHR) is a pivotal enzyme within the isoflavonoid biosynthetic pathway and is classified under the AKR4 subfamily, which forms part of the broader AKR superfamily [34]. The CHR protein catalyzes the reduction of various carbonyl compounds but requires NADPH as an essential cofactor. Working in concert with chalcone synthase (CHS), CHR channels the substrates p-coumaroyl-CoA and malonyl-CoA toward the production of isoliquiritigenin. When acting alone, however, CHS directs the same substrate pair to form naringenin chalcone [35]. Type II chalcone isomerase (CHI), a characteristic enzyme found in leguminous plants, mediates the isomerization of naringenin chalcone to naringenin and of isoliquiritigenin to liquiritigenin [36]. IFS, a cytochrome P450 monooxygenase, executes a crucial aryl migration reaction, thereby generating the core isoflavone structures of genistein (5,7,4′-trihydroxyisoflavone) and daidzein (7,4′-dihydroxyisoflavone). As the entry-point enzyme of the isoflavone-specific branch, IFS plays a decisive role in determining the metabolic flux toward isoflavonoids [37]. In this study, we identified the protein products encoded by *AmCHR*, *AmCHS*, *AmCHI*, and *AmIFS* genes and verified their catalytic functions, as well as their roles in the isoflavonoid biosynthetic pathway of *A. mongholicus*. We demonstrated that the AmCHS protein from *A. mongholicus* exhibits Claisen condensation activity, and when the AmCHR protein was added to the reaction system, the catalytic product shifted to isoliquiritigenin. Furthermore, functional characterization of *AmIFS* using a yeast heterologous expression system revealed its capacity to catalyze the conversion of both naringenin to genistein and liquiritigenin to daidzein. These findings not only elucidate the catalytic mechanisms of key enzymes in the isoflavonoid pathway but also provide essential genetic modules for the future metabolic engineering and ‘green synthesis’ of bioactive isoflavonoids.

For perennial medicinal plants, it is difficult to ensure their normal growth when cultivated in the field, resulting in low accumulation of effective components, and they are easily affected by environmental changes, pests and diseases, pesticide residues, and other adverse effects. As a result, the quality of medicinal plant raw materials is inconsistent, and it is difficult to meet the high demand of domestic and foreign markets and strict quality standards [38]. To circumvent these limitations, plant cell and tissue culture technologies have emerged as pivotal strategies for the sustainable, large-scale production of specialized metabolites [39]*. Agrobacterium rhizogenes*–mediated transformation technology has promoted the development of the hairy root culture system. Owing to its rapid growth and biochemical and genetic stability, it has attracted significant attention as a safe, reliable, genetically stable, rapid growth, large-scale production method that has high synthesis ability of secondary metabolites and affords easy preservation [40]. Recent advances in *A. membranaceus* hairy root cultures have demonstrated their potential for industrial-scale metabolite production [41]. However, the application of hairy roots in *A. membranaceus* has been limited because of the high operational requirements and tedious tissue culture process required by existing transformation technologies. Building upon recent biotechnological breakthroughs, a streamlined, *ex vitro* hairy root transformation method that bypasses the need for sterile conditions and complex regeneration steps has been successfully implemented in various medicinal species, including *A. membranaceus*, *Scutellaria baicalensis*, and *Codonopsis pilosula* [42]. Nevertheless, this efficient technology has yet to be extensively integrated into the genetic improvement and functional genomics of *A. mongholicus*. This study established a simplified hairy root transformation system for *A. mongholicus* and elucidated its potential application value for producing secondary metabolites in medicinal plants. This system does not require complex tissue culture procedures or a sterile environment, yet it yields hairy roots with high survival rates and excellent transformation efficiency. In addition to employing the classic *EGFP* and *PAT* reporter genes, this study innovatively introduced the *RUBY* reporter gene, enabling effective distinction between wild-type and transgenic hairy root lines. Compared with the traditional *EGFP* reporter, the *RUBY* reporter is more convenient to use and provides more visually intuitive results. Furthermore, *RUBY* facilitates nondestructive monitoring of gene expression and offers a more eco-friendly and biosafe alternative to traditional antibiotic or herbicide resistance markers [43]. However, it should be noted that the transformation efficiency of *RUBY* (26.7%) was considerably lower than that of *EGFP* (75%) and *PAT* (66.7%). This observation is consistent with recent findings in pepper transformation studies, where comparative analysis of three reporter genes (*DsRed*, *EGFP*, and *RUBY*) revealed significant differences in their transformation efficiencies, with *RUBY* achieving only ~5% stable transformation efficiency despite its superior visual screening capability [44]. These results suggest that different reporter genes may exhibit variable expression efficiencies depending on the transformation system and target species and that the larger construct size or specific expression requirements of *RUBY* may contribute to its relatively lower transformation efficiency. Collectively, our findings demonstrate that this optimized genetic transformation system establishes a stable and highly reproducible platform for the induction of transgenic hairy roots in *A. mongholicus*, facilitating future functional genomics and metabolic engineering in this medicinal species.

The functional roles of key biosynthetic enzymes within the isoflavonoid pathway have been extensively characterized in the model legume *Glycine max* [45]. For instance, the heterologous expression of the soybean *GmIFS* gene in *Arabidopsis thaliana* resulted in genistein production, demonstrating the enzyme’s capacity to drive isoflavonoid synthesis in nonleguminous species and confirming its fundamental role in the pathway [46]. The soybean *GmCHI4s* genes are predominantly expressed in roots. Hairy root plants overexpressing *GmCHI4A* and GmCHI4B showed 6-fold and 3-fold increases in total isoflavonoid content, respectively. In *GmCHI4A* overexpressing hairy roots, daidzein and glycitein were significantly accumulated, whereas *GmCHI4B* overexpressing lines exhibited markedly elevated levels of daidzein, genistin, glycitein, and biochanin A. These findings identify *GmCHI4* genes as pivotal components of the soybean isoflavonoid pathway [47]. However, analogous research in nonmodel medicinal plants remains comparatively sparse. Leveraging our established nonsterile transformation system, we demonstrated that the overexpression of *AmCHR*, *AmCHS*, *AmCHI*, and *AmIFS* significantly augmented the accumulation of CA, CAG, FO, and ON in *A. mongholicus*, whereas RNAi-mediated attenuation of these genes produced the reciprocal metabolic phenotype. Using the established nonsterile hairy root genetic transformation system, overexpression of *AmCHR*, *AmCHS*, *AmCHI*, and *AmIFS* significantly increased the accumulation of CA, CAG, FO, and ON in *A. mongholicus* hairy roots, whereas RNAi experiments yielded opposite results. The results from AsODN-mediated transient silencing further supported this experimental outcome. These results confirm that *AmCHR*, *AmCHS*, *AmCHI*, and *AmIFS* are key enzyme genes for isoflavonoid biosynthesis in *A. mongholicus*. In summary, these findings not only corroborate previous investigations into the functions of these genes but also provide a referential research paradigm for studying the isoflavonoid biosynthetic pathway in medicinal plants while offering candidate gene resources for molecular assisted breeding of high-quality medicinal materials.

## Conclusion

**Figure 8:**
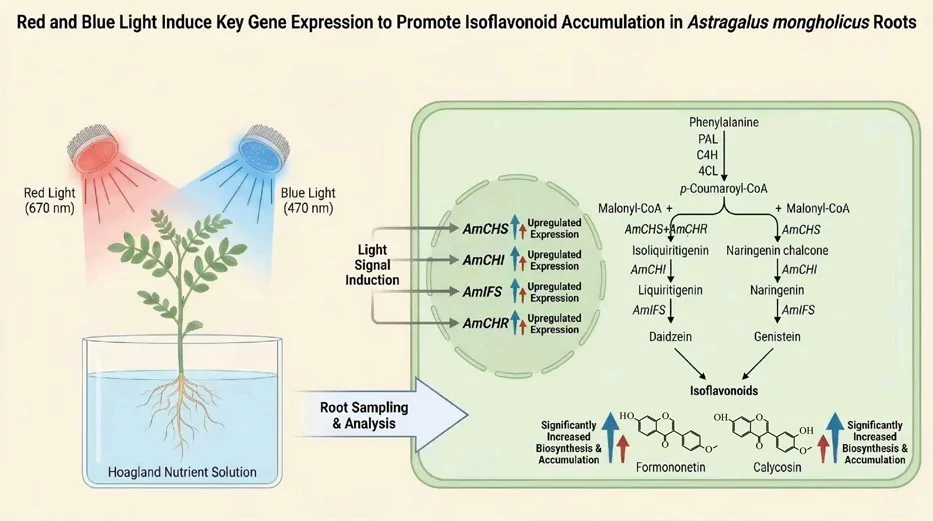
A working model illustrating how red and blue light promote isoflavonoid biosynthesis in the roots of *A. mongholicus*. Red and blue light induce the expression of *AmCHR*, *AmCHS*, *AmCHI,* and *AmIFS*, the key genes involved in isoflavonoid biosynthesis, thereby promoting the accumulation of isoflavonoids including CA and FO; among them, the promoting effect of blue light is significantly superior to that of red light.

This study deciphered the molecular mechanisms by which different light qualities regulate isoflavonoid biosynthesis in *A. mongholicus* through integrated transcriptomic and metabolomic analyses. The results demonstrate that blue light more effectively promotes the accumulation of the key medicinal components CA and FO than red light, and this light quality–specific regulation is closely associated with the expression of four core genes: *AmCHR*, *AmCHS*, *AmCHI*, and *AmIFS*. The catalytic functions of these proteins were confirmed through *in vitro* enzymatic assays, and their positive regulatory roles in isoflavonoid accumulation were further validated *in vivo* using hairy root genetic transformation and transient gene silencing techniques. Among them, *AmCHI* and *AmIFS* exhibited more pronounced regulatory effects, suggesting that they may serve as key rate-limiting enzymes in the isoflavonoid biosynthetic pathway. (Fig. 8). These findings not only reveal the molecular basis of isoflavonoid biosynthesis in *A. mongholicus* but also provide candidate genes for the green biosynthesis of medicinal isoflavonoids. Future investigations should focus on the upstream regulatory factors, such as light-responsive transcription factors and their interactions with primary metabolism to fully unravel the complex regulatory network orchestrating isoflavonoid biosynthesis in this important medicinal plant.

## Materials and methods

### Plant material and measurements

Seeds of *A. mongolicus* employed in this experiment were commercially sourced from the Anguo medicinal material market. Their botanical identity was confirmed by Researcher Ma Wei (Heilongjiang University of Chinese Medicine). Seed germination was induced by soaking. Seeds selected for uniformity, plumpness, and integrity underwent surface decontamination using 75% ethanol (30 s), followed by three rinses with sterile distilled water and subsequent drying on sterile filter paper. The seeds were then placed in Petri dishes for soaking. Germination was carried out in darkness on filter paper saturated with distilled water. The filter paper was replaced with fresh saturated paper every 24 h. The germinated seedlings were cultivated hydroponically and subjected to different light treatments: white light (5000 K LED, simulating normal light), monochromatic blue light (470 nm), or monochromatic red light (670 nm). All treatments maintained a photosynthetic photon flux density of 50 ± 5 μmol·m^−2^·s^−1^, with a photoperiod cycle of 16-h light and 8-h darkness. Plants were cultivated under controlled environmental conditions: a daytime/nighttime temperature regime of 25/20°C, relative humidity at 60%, and were regularly irrigated with a modified Hoagland nutrient solution (Beijing Coolaber Science & Technology Co., Ltd.) throughout the 30-day experimental period. Following the treatment period, roots harvested from 10 uniformly developed plants within each group were combined to constitute a single biological replicate. To minimize circadian rhythm effects, all samples were collected at the same time of day. Samples were collected randomly from independent batches of plants grown under identical conditions. Each biological replicate was derived from a separate batch of plants to ensure true biological independence. Each treatment group comprised three such independent biological replicates. Samples were snap-frozen in liquid nitrogen and stored for subsequent transcriptomic, metabolomic, qRT-PCR, and gene cloning analyses.

### Transcriptome sequencing

Root tissues of *A. mongolicus* were subjected to total RNA isolation, employing a CTAB-PBIOZOL reagent kit. Messenger RNA containing polyadenylated tails was subsequently purified using oligo(dT) magnetic beads, chemically fragmented, and then used as template for first- and second-strand cDNA synthesis. The resulting cDNA underwent end repair, adapter ligation, and fragment size selection before being amplified by PCR. Final libraries were sequenced on an Illumina sequencer to generate 150-bp paired-end reads. Raw sequencing data were processed with fastp [48] software to remove adapter-containing reads, reads with N content >10%, and low-quality reads (Q ≤ 20) exceeding 50% of the read length, yielding high-quality clean reads. Clean reads were aligned to the reference genome using HISAT2 software [49]. Quality control criteria required Q30 > 85% and mapping rate > 80% for all samples.

### Metabolomic profiling

Metabolite extraction was conducted for all nine experimental samples, representing three light treatments each with three biological replicates. Following freeze-drying, the samples were pulverized into a homogeneous powder with a mixer mill. Precisely 50 mg of the powdered material was then weighed and extracted with 1200 μl of pre-chilled (−20°C) 70% methanol–water solution. The mixture was subjected to ultrasonic extraction for 30 min. This extraction process was repeated three times to maximize metabolite recovery. The supernatants from each round were combined after centrifugation. The pooled extracts were then vacuum-dried to complete dryness. The residue was redissolved in 100 μl of 70% methanol in preparation for liquid chromatography–mass spectrometry (LC-MS) analysis. LC-MS analysis was carried out on an ultra-performance liquid chromatography (UPLC) system (ExionLC™ AD, Sciex) coupled with an electrospray ionization tandem mass spectrometry (ESI-MS/MS) system [50]. Metabolite identification was achieved by matching the acquired MS/MS spectra against the proprietary standard compound database maintained by MetWare Co., Ltd. (Wuhan, China). Before injection, the extract was filtered through a 0.22-μm microporous membrane. Chromatographic separation was performed on an Agilent SB-C18 column (1.8 μm, 2.1 mm × 100 mm) at 40°C with a mobile phase consisting of 0.1% formic acid in water (A) and 0.1% formic acid in acetonitrile (B). The gradient program was as follows: 0 min, 95% A/5% B; 0–9 min, linear gradient to 5% A/95% B; 9–10 min, hold at 5% A/95% B; 10–11.1 min, linear gradient to 95% A/5% B; 11.1–14 min, hold at 95% A/5% B. The flow rate was 0.35 ml/min, and the injection volume was 2 μl. The ESI source parameters were set as follows: source temperature 500°C; ion spray voltage 5500 V (positive ion mode) and 4500 V (negative ion mode); ion source gas I, gas II, and curtain gas set at 50, 60, and 25 psi, respectively; collision-activated dissociation was set to high. QQQ scans were acquired in multiple reaction monitoring mode.

### Statistical analysis

The expression abundance of each gene was calculated and normalized using the FPKM metric. To identify genes with statistically significant expression changes across light treatments, differential expression analysis was performed utilizing the DESeq2 software package in R [51]. To account for the increased risk of false positives arising from multiple comparisons, the raw *P*-values were adjusted to control the false discovery rate (FDR) via the Benjamini–Hochberg method. DEGs were identified with the thresholds of |log₂Fold Change| ≥ 0.8 and FDR < 0.05.

The normalized metabolomic data were imported into the MetWare Cloud Platform for principal component analysis (PCA) to evaluate intersample metabolite differences and intragroup variability. Orthogonal Partial Least Squares Discriminant Analysis (OPLS-DA) was then employed to further distinguish intergroup differences, with a variable importance in projection (VIP) > 1 considered statistically discriminative. Differentially accumulated metabolites (DAMs) were screened by integrating VIP values and fold change, using the criteria VIP > 1 and fold change ≥1.8 or FC ≤ 0.5.

To visualize the interaction patterns between transcriptional changes and metabolite accumulation, a correlation network was generated and rendered using Cytoscape (version 3.7.1) [52]. A co-expression network was built to elucidate gene–metabolite relationships. This was achieved by calculating pairwise correlations between the FPKM values of DEGs annotated to the isoflavonoid biosynthetic pathways and the relative abundance levels of differentially accumulated isoflavonoids [53]. Prior to analysis, both the gene FPKM data (transcriptome) and metabolite relative abundance data (metabolome) were log2-transformed to approximate normal distribution. Associations were then assessed by computing the Pearson correlation coefficient for each gene-metabolite pair based on nine samples (three biological replicates per treatment group). The correlation threshold was set at |*r*| > 0.9 with a *P*-value < 0.05. To account for multiple testing, *P*-values were adjusted using the Benjamini–Hochberg method to control the FDR, with adjusted *P* < 0.05 considered statistically significant.

### qRT-PCR

To independently verify the transcriptome sequencing results, the expression levels of selected genes were quantified using qRT-PCR following standard molecular biology procedures. The workflow included total RNA extraction, synthesis of first-strand complementary DNA, amplification of target sequences, and final quantification analysis [54]. The *18S* gene was utilized as an internal control for normalizing expression data. Each qRT-PCR assay was run in triplicate technical replicates for each of the three independent biological replicates per treatment group. qRT-PCR data are presented as mean ± SD of three technical replicates. Statistical analysis was performed using Student’s *t*-test. The sequences of all gene-specific primers designed are provided in Supplementary Table S7.

### Subcellular localization

The ORFs of *AmCHR*, *AmCHS*, *AmCHI*, and *AmIFS* were individually inserted into the *MluI* restriction site of the pCAMBIA1302 vector carrying an enhanced green fluorescent protein (EGFP) tag using the ClonExpress Ultra One Step Cloning Kit V2 (Vazyme Biotech Co. Ltd., China). Subsequently, the recombinant vectors were transformed into *A. tumefaciens* EHA105 competent cells (Weidi Biotechnology Co., Ltd., Shanghai, China). Positive transformants were selected on Luria-Bertani (LB) agar plates containing kanamycin (50 mg/L) and rifampicin (50 mg/L). The bacterial cells were subsequently resuspended in infection buffer (10 mM MgCl₂, 10 mM 2-(N-morpholino)ethanesulfonic acid (MES), 100 μM acetosyringone, pH 5.8) and adjusted to an OD600 = 0.4. For transient expression and subcellular localization observation, the adjusted bacterial suspensions were infiltrated into the abaxial (lower) epidermis of fully expanded leaves from 30-d *N. benthamiana* plants using a 1-ml needleless syringe. Postinfiltration, the plants were maintained in darkness for 24 h to facilitate bacterial infection and gene transfer, before being transferred to a controlled growth chamber (25°C, 16-h light/8-h dark cycle) for an additional 48 h to allow for transgene expression. Finally, green fluorescent signals were observed using LSM800 with Airyscan (Carl Zeiss, Oberkochen, Germany).

### Bacterial expression and *in vitro* characterization

The full-length ORFs of *AmCHR*, *AmCHS*, and *AmIFS* were individually cloned into the *HindIII* restriction site of the pET-28a expression vector. The resulting recombinant plasmids were then transformed into *E. coli* BL21 (DE3) competent cells. Recombinant protein expression was induced by adding IPTG to a final concentration of 0.5 mM. The culture was then continued under shaking for an additional 8 h at the prescribed temperature to allow protein production [55]. To determine the optimal induction temperature, protein expression was evaluated across a temperature range of 16, 20, 25, 30, and 37°C. After induction, cells were pelleted by centrifugation at 5000 × *g* for 10 min at 4°C. The cell pellets were stored at −80°C or processed immediately for protein purification using a commercial Ni-NTA affinity chromatography kit (Beyotime Biotechnology) according to the manufacturer’s instructions.

For size-based separation, recombinant protein samples were subjected to SDS-PAGE using hand-cast gels with a 5% stacking gel and a 12% resolving gel. Gels were stained with Coomassie Brilliant Blue to determine the optimal expression temperature. WB analysis was further performed to validate the proteins encoded by the candidate genes. Proteins were transferred onto polyvinylidene difluoride (PVDF) membranes that had been pre-activated with methanol. For immunodetection, membranes were probed with a mouse-derived monoclonal primary antibody against the Strep-tag II epitope. A horseradish peroxidase–conjugated goat anti-mouse IgG antibody was then applied as the secondary antibody. Membranes were treated with ECL chemiluminescent substrate, and signals were finally visualized and imaged using a gel imaging system. All antibodies and reagents for western blotting were purchased from Bioss Biotechnology (Beijing, China).

*In vitro* enzymatic activity assays were performed in a total reaction volume of 2.53 μl. Each reaction contained ~0.5 μg of purified recombinant protein (in 10 mM Tris-HCl buffer, pH 7.5) and 1 mM of the respective substrate. For the CHR protein activity assay, the reaction mixture was supplemented by adding an equimolar amount of CHS protein and 1 mM NADPH as an essential cofactor. All enzymatic reactions were carried out at 30°C for 1 h in a thermal cycler or water bath, after which they were stopped by adding 200 μl of ice-cold ethyl acetate. The organic phase containing the reaction products was collected, dried under a gentle nitrogen stream, redissolved in a suitable solvent, and subsequently subjected to analysis by high-performance liquid chromatography (HPLC) [56].

### Yeast expression and *in vivo* characterization

The full-length ORF of the cytochrome P450 gene *AmIFS* was cloned into the pESC-URA vector at the *SmaI* and *SalI* restriction sites. The recombinant plasmid was then transformed into the WAT11 yeast strain. Transformants were selected by plating on synthetic complete medium lacking uracil (SC-URA), supplemented with 2% (w/v) glucose and solidified with 1% (w/v) agar. Plates were incubated at 29°C for 3 days to allow colony formation. A single positive colony was inoculated into 10 ml of liquid SC-URA medium (with 2% glucose) as a starter culture. This preculture was grown overnight at 29°C with orbital shaking at 200 rpm. The overnight culture was then diluted by transferring 5 ml into 200 ml of fresh SC-URA liquid medium (with 2% glucose) in a flask. Cells were grown under the same conditions (29°C, 200 rpm) until the mid-log phase, corresponding to an optical density at 600 nm (OD600) of ~0.8. Yeast cells were harvested by centrifugation (5000 × *g*, 5 min, 4°C). The cell pellet was washed three times with 10 ml of sterile distilled water to remove residual glucose and finally resuspended in 100 ml of induction medium (SC-URA containing 2% galactose as the carbon source). Gene expression was induced by culturing the resuspended cells at 29°C with vigorous shaking (160 rpm) for 6 h. Following induction, the appropriate substrates (liquiritigenin or naringenin) were added to the culture to a final concentration of 100 μM each. The substrate-fed cultures were then incubated for an additional 12 h under the same conditions to allow for bioconversion. After the incubation period, the entire culture broth was collected. Metabolites were extracted by partitioning three times against an equal volume of ethyl acetate. The pooled ethyl acetate phases were combined and concentrated to complete dryness under reduced pressure at 30°C using a rotary evaporator. The dried extract was reconstituted in a defined volume of HPLC-grade methanol. This final sample solution was filtered through a 0.22-μm syringe filter for clarification and then subjected to HPLC analysis.

### *A. rhizogenes* mediated genetic transformation under nonsterile conditions

Following germination, the *A. mongolicus* seedlings were transferred to a standard potting substrate and cultivated in a controlled growth chamber. The environmental settings were maintained at a 25/20°C (day/night) thermoperiod, 60% relative humidity, and a photoperiod of 16 h of light followed by 8 h of darkness. After 21 days of cultivation, vigorous and disease-free *A. mongholicus* plants were selected for genetic transformation experiments. The transformation vector was constructed based on the binary plasmid pCAMBIA1300, using either the CaMV 35S promoter-driven *EGFP* or the *RUBY* as the reporter, along with the MAS promoter-driven *PAT* gene from vector PFCG5941 as an additional reporter. The recombinant plasmids were introduced into *A. rhizogenes* strain K599. Transformants carrying the *EGFP* and *PAT* reporter genes were inoculated into TY medium supplemented with 50 mg/L streptomycin and 50 mg/L kanamycin, while transformants carrying the *RUBY* reporter gene were inoculated into TY medium containing 50 mg/L streptomycin and 100 mg/L spectinomycin. Cultures were incubated with shaking at 28°C and 200 rpm until the OD_600_ = 0.6–0.8. The bacterial suspensions were then resuspended in MES buffer to a final OD_600_ = 0.6. This suspension was then pre-induced by incubating at 28°C with gentle shaking (50–80 rpm) for 2 h to activate the *vir* genes prior to plant infection. Twenty-one-day *A. mongholicus* seedlings were gently uprooted, and their root systems were thoroughly yet gently rinsed to eliminate adhering soil particles. To prepare explants for transformation, the roots and a portion of the leaves were aseptically removed, leaving the hypocotyl region intact*.* The prepared explants were immersed in the resuspension solution and kept in darkness for 30 min. The explants were then inserted into seedling trays containing moist vermiculite, and 5 ml of the resuspension solution was applied along the stem of each explant until it reached the base. The trays containing the infected explants were first maintained in complete darkness for 48 h. Subsequently, they were transferred to low-intensity light conditions for 7 days to allow initial recovery and transgenic cell proliferation, before finally being moved to standard growth light conditions. Throughout all postinfection culture stages, the environmental conditions were consistently maintained at a temperature of 25°C with a 16-h light/8-h dark photoperiod.

After the hairy roots had grown for ~30 days, the plants were gently flushed with water to remove them from the vermiculite, minimizing damage to the hairy roots. Hairy roots transformed with the *EGFP* were visualized by green fluorescence excited with a LUYOR-3415 light source [57]. Positive hairy roots carrying the *PAT* gene were detected using Bar test strips. For constructs harboring the *RUBY* reporter gene, which drives the production of the red pigment betalain, positive transgenic hairy roots were readily distinguishable by their vivid red coloration, enabling visual screening without any specialized equipment or chemical assays. To molecularly confirm transgenic events, genomic DNA was isolated from a subset of putative positive hairy root lines using the CTAB method. PCR was performed using 1 μl of diluted DNA as template with gene-specific primers for *EGFP*, *RUBY*, and *PAT*. Successful amplification of the expected fragments provided definitive evidence for the genomic integration of the respective transgenes [58]. The transformation efficiency of different reporter genes was statistically analyzed to evaluate the stability of the transgenic hairy root system in *A. mongholicus*. The efficiency of the transformation system was calculated using the following formulas: Survival rate = (Number of surviving plants/Total number of infected plants) × 100%; Transformation rate = (Number of positive plants/Total number of infected plants) × 100% [59].

### Overexpression and RNAi

The ORF of *AmCHR*, *AmCHS*, *AmCHI*, and *AmIFS* genes were individually inserted into the *SpeI* restriction site of the pCAMBIA1300-UBi-MCS-35S-EGFP vector. The recombinant vectors were then transformed into strain K599 for overexpression experiments. For RNAi experiments, the sense and antisense strands of a 300-bp candidate gene-specific fragment were inserted between the *SwaI* and *XbaI* restriction sites of the plant RNAi vector pFGC5941 (Supplementary Table S8), in accordance with its design features [60]. *AmCHR*, *AmCHS*, *AmCHI*, and *AmIFS* genes specific fragments were screened using the online SGN VIGS Tool [61].

### Gene suppression in *A. mongholicus* leaves using AsODN

The sODN sequences matching candidate gene fragments and their reverse complement AsODN sequences were designed using the Soligo software [62]. To prevent nuclease degradation, the three terminal bases at both ends of the primers were phosphorothioate-modified. The AsODN and sODN primers for the candidate genes were diluted to 5 μM with ddH₂O. To deliver the oligonucleotides, the root systems of 21-day *A. mongholicus* seedlings were first carefully excised. The whole seedlings were then immersed in the respective oligonucleotide solutions and incubated in complete darkness for 48 h to facilitate uptake. Seedlings treated with the corresponding sODN solutions for each gene were included as negative controls. Following the incubation period, 10 uniformly sized seedlings were harvested per treatment group to constitute one biological replicate. The entire experiment was independently performed three times to ensure statistical robustness.

### Determination of isoflavonoid compounds

Exactly 50 mg of freeze-dried transgenic hairy roots powder or 100 mg of fresh leaf tissue from AsODN treated *A. mongholicus* plants was weighed and extracted by ultrasonication with 70% methanol for 1 h. The crude extracts were then clarified by passing through a 0.22-μm syringe filter. The filtrate was collected directly into certified HPLC vials in preparation for instrumental analysis. Quantitative analysis of isoflavonoids was performed on an ultra-high-performance liquid chromatography coupled with quadrupole time-of-flight mass spectrometry platform (Shimadzu LCMS-9030). The mobile phase consisted of two components: (i) ultrapure water containing 0.1% (v/v) formic acid and (ii) mass spectrometry–grade acetonitrile. The following gradient elution program was used for mobile phase B: 0–2 min, 5%–40%; 2–8 min, 40%–70%; 8–10 min, 70%–95%; 10–14 min, 95% back to 5%. All detection experiments were performed in triplicate. CA, FO, CAG, and ON standards (purity >98%) were used for quantification (Sichuan weikeqi Biological Technology Co., Ltd. Chengdu., Chengdu, China).

## Supplementary Material

Web_Material_uhag192

## Data Availability

The raw RNA-seq data have been deposited in the Sequence Read Archive (http://www.ncbi.nlm.nih.gov/sra/) under accession number PRJNA1064679 and PRJNA1415693.
