# Supplementary material for: Integrated transcriptomic and metabolomic analysis unveils biosynthetic mechanisms of key bioactive isoflavonoid accumulation in Astragalus mongholicus under different light treatments
Source: Hortic Res. 2026 May 20;13(10):uhag192. doi: 10.1093/hr/uhag192 (PMC13616494; doi:10.1093/hr/uhag192)
Supplement: Supplementary file 1 [file web_material_uhag192.zip › Figure s1-s4.docx]

**Supplementary figures**

**Figure S1.** Transcriptomic analysis reveals the effects of different light treatments on *A. Mongholicus.* (A) Volcano plot analysis of differentially expressed genes (DEGs) comparing White (CK), Red (R) and Blue (B) light treatments. ( B) Principal Component Analysis (PCA) of RNA-Seq samples.

**Figure S2.** Non-sterile *A. rhizogenes*-mediated hairy root transformation system for *A. mongholicus.*(a) Intact whole seedlings of *A. mongholicus.*(b) Explants prepared for inoculation by cutting the hypocotyls. (c) The hairy roots carrying the EGFP report gene emitted green fluorescence upon excitation with blue light.

**Figure S3.** LC-MS/MS analysis of AmIFS reaction products. (A-B) AmIFS catalytic conversion of 1 to 2 and the products were analyzed by HPLC and MS. (C-D) AmIFS catalytic conversion of 1 to 2 and the products were analyzed by HPLC and MS. 1, 2, 3, and 4 correspond to liquiritigenin, daidzein, naringenin, and genistein, respectively

**Figure S4.** Distribution of *cis*-regulatory elements in the promoter regions of four core isoflavonoid biosynthetic genes. The promoter sequences (2000 bp upstream of the start codon) are shown as horizontal bars. Different *cis*-elements are represented by colored symbols according to the legend.
